# Metabolic control and its associated factors in type 1 diabetic people: longitudinal trajectory modeling

**DOI:** 10.1186/s12889-024-19098-1

**Published:** 2025-02-03

**Authors:** Zahra Khosravi, Ali Taghipour, Monavar Afzalaghaee, Habibollah Esmaily, Ahmad Khosravi

**Affiliations:** 1 Department of Epidemiology, School of Health, Mashhad University of Medical Sciences, Mashhad, Iran; 2 Social Determinants of Health Research Center, Mashhad University of Medical Sciences, Mashhad, Iran; 3 Department of Biostatistics, School of Health, Mashhad University of Medical Sciences, Mashhad, Iran; 4 Department of Epidemiology, School of Public Health, Shahroud University of Medical Sciences, Shahroud, Iran

**Keywords:** Type 1 diabetes, Glycosylated hemoglobin, Latent class growth modeling

## Abstract

**Background:**

Diabetes is a chronic disease, and hyperglycemia can increase the risk of diabetic complications and the need for more inpatient services. Therefore, the prevention and control of diabetes are important. This study aimed to identify the trajectories of metabolic control and its correlates in people with type 1 diabetes.

**Method:**

This is a longitudinal study with 2020 type 1 diabetic individuals aged 18 to 59 years. The participants’ glycosylated hemoglobin (HbA_1c_) was measured three times with a six-month interval between each measurement. The data were analyzed using group-based trajectory modeling. Multinomial logistic regression was used to determine the factors related to these groups.

**Results:**

The results showed four trajectories of safe controlled (46.2%), moderate stable risk (28.7%), moderate increasing risk (12.5%), and high decreasing risk trajectory (12.6%) (entropy = 0.70). The results of multinomial logistic regression showed dyslipidemia could increase the odds of being in the three risk trajectories. Education, physical inactivity, and poor psychological status could also increase the odds of being in the moderate stable and high decreasing trajectories. Moreover, sex, job, and BMI could increase the odds of being in the high decreasing risk group (*p* < 0.05).

**Conclusion:**

Since there are different trajectories of metabolic control of diabetes, it is necessary for healthcare providers and health experts to plan behavioral interventions based on the location of individuals in different trajectories and the related significant risk factors. In this way, appropriate prevention, care, and treatment programs can be provided for the people in each group.

## Background

Diabetes mellitus is characterized by impaired metabolism of carbohydrates, fats, and proteins, which is associated with an absolute or relative lack of insulin [1]. The disease can appear in the forms of type 1, type 2, gestational, and secondary diabetes. Type 2 diabetes is the most common form of diabetes. However, due to its development in childhood and lack of definitive treatment, type 1 diabetes is of paramount importance [2]. The total number of people with diabetes in the world is predicted to increase from 366 million in 2011 to 552 million in 2030 [3]. In addition, it is estimated that the number of people with diabetes in Iran will increase from 3.78 million in 2009 to 9.24 million in 2030, and the number of deaths caused by all diabetes forms in Iran will increase from 38,000 in 2009 to 89,000 in 2030 [4].

The incidence of type 1 diabetes is increasing in different countries. In children under 14, the annual incidence varies from 0.1 per 100,000 in China and Venezuela to 36.5 per 100,000 in Finland. The highest incidence was observed in Sardinia, Finland, Norway, Sweden, England, Canada, Portugal, and New Zealand, and the lowest incidence was observed in China and South America [5]. According to a review study in 2019, the incidence of type 1 diabetes in the world was 15 per 100,000 people, and its prevalence was reported to be 9.5 per 10,000 people [6].

Zamanfar and colleagues, in a study of type 1 diabetes in the northern province of Iran, reported an incidence of 48 cases per 100,000 people aged 7 months to 18 years [7]. In southern Iran, its incidence has been reported at 3.7 per 100,000 people [8]. In most populations, the prevalence has increased with age and has been the highest among children aged 10–14 years [5].

Due to its chronic course and effect on other metabolic factors, such as cholesterol, triglycerides, and atherosclerotic agents, the diagnosis and control of diabetes is very important. To control blood sugar and other metabolic factors, a person needs insulin throughout life. If glucose is not controlled or poorly cared for, it can increase the risk of diabetic retinopathy and nephropathy, as well as skeletal and muscular complications, and there will be more need for inpatient services. Moreover, these complications can lead to job loss and increase the direct and indirect costs of treatment [9, 10].

One of the most important factors in reducing the complications of the disease is performing self-care behaviors that include a range of activities, such as blood sugar control, a diet without trans fatty acids, physical activity, daily checking of legs and feet, and taking medication. Low self-efficacy and depression, however, reduce adherence to self-care behaviors [11]. The best way to assess the therapeutic response to control measures is to check HbA_1c_ in the last 3 months. According to the International Diabetes Federation, the goal of controlling diabetes is HbA_1c_ less than 7% for all age groups [12]. In fact, adherence to the principles of diabetic self-care leads to a decrease in hemoglobin HbA_1c_, control of blood sugar, and reduction of complications such as ketoacidosis, retinopathy, nephropathy, neuropathy, and macrovascular complications of diabetes [13].

There are different statistical approaches for estimating the response to treatment over time, the two most important of which are repeated measures multivariate analysis of variance, and structural equation modeling. However, the premise of these approaches is that all people follow the same path or trajectory over time [14, 15]. Since in diabetes, there may be unknown groups of people hidden in the population who follow different patterns that are different in the strength and direction of the changes, repeated measures methods may not be appropriate. However, one of the approaches to latent class growth modeling (LCGM) is group-based trajectory modeling (GBTM). This method can categorize people who follow different patterns in the variable under study over time [15, 16]. In this model, several averages are specified for hidden classes or groups, and differences between groups are shown. Individuals in each group are similar in terms of changes in the variable over time, all individual growth trajectories in a group are the same, and variables that affect growth factors affect individuals in the same group equally [17].

GBTM is in fact useful in identifying vulnerable populations that need better health care as well as identifying trajectories that lead to the best health outcomes. Such a new approach to epidemiology could provide stronger scientific evidence for optimizing health care focused on the needs of specific subgroups. In addition, by identifying different trajectories and groups and placing a person in one of the groups, the degree of his vulnerability to diabetes is revealed, and therefore, it is possible to plan better prevention, care, and treatment programs according to the factors associated with each group.

However, all domestic studies on the incidence and prevalence of diabetes in Iran are cross-sectional, and the vast majority of foreign studies conducted longitudinally have used standard growth methods to analyze their data. Therefore, this study aimed to use GBTM and identify people with diabetes who follow similar trajectories in glycosylated hemoglobin (HbA_1C_). It further aimed to examine the metabolic control changes in these individuals and divide them into categories with similar characteristics based on the pattern they had over this time. The study also aimed to investigate the factors associated with each of the identified trajectories.

### Method

This study is a longitudinal study with secondary data. The data used in this study were obtained from the SINA Electronic Health Records of Mashhad University of Medical Sciences. The data in the SINA are collected from people who are cared for in comprehensive healthcare centers located in different districts and neighborhoods of the city. People are encouraged to refer to these healthcare centers for various public health purposes such as pregnancy care, baby and child care, vaccination, diabetes care, hypertension care, etc. People in different areas of the city with diverse socioeconomic statuses voluntarily refer to health care centers for checkups and monitoring and are examined by physicians and health care personnel, and their data are registered on the SINA Health Records. Due to the large number of people from various backgrounds that receive primary health care services in these centers, the data on the SINA are almost representative of all the population in the city.

**Fig. 1:**
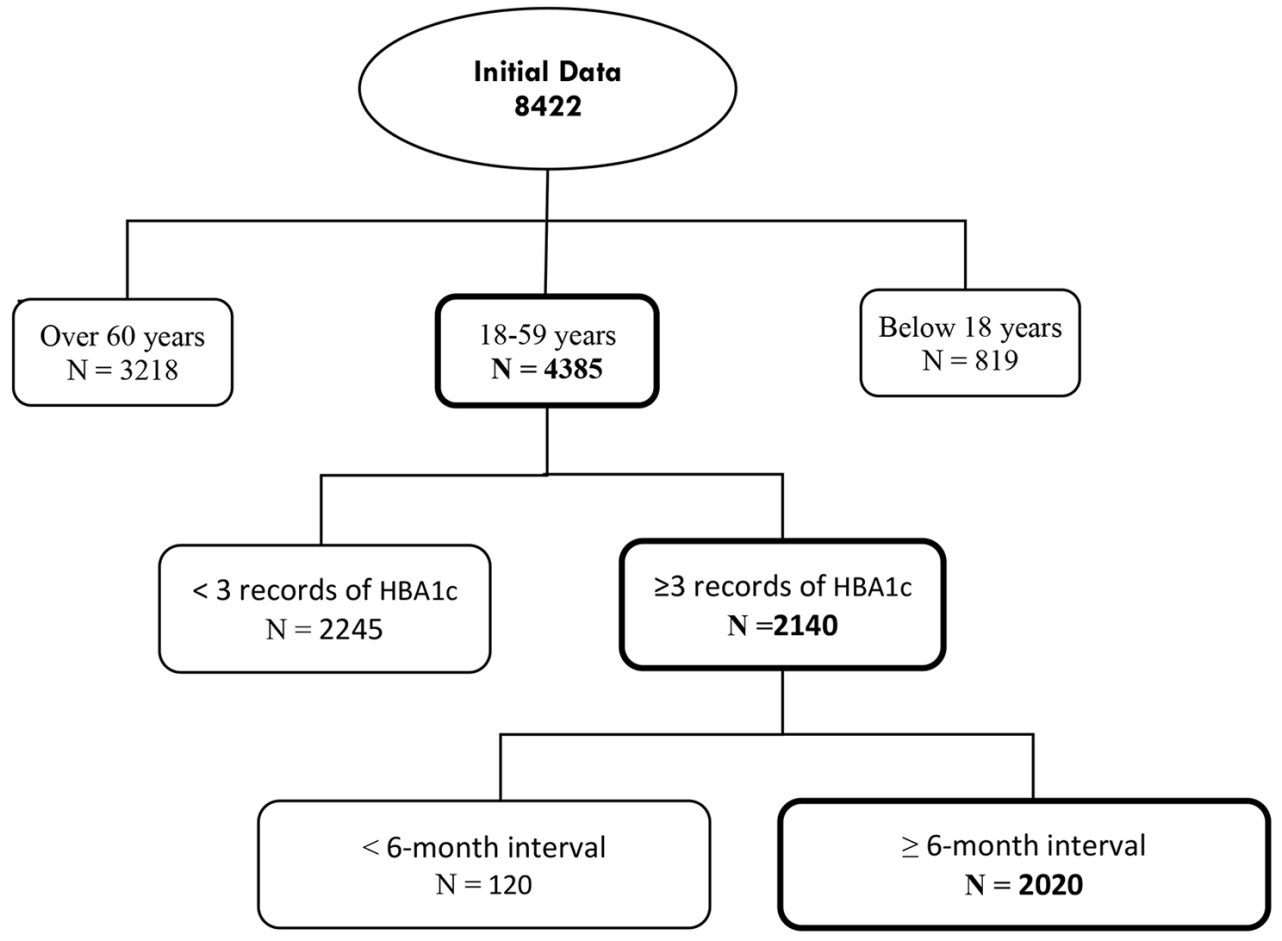
Flow chart of data selection process

In line with the requirement of the latent class modeling approaches which require at least three measurement time points for longitudinal studies [15, 17], all individuals aged 18 to 59 years old who were identified and registered with type 1 diabetes mellitus since 2016 and whose glycosylated hemoglobin was measured three or more times with an interval of 6 months (*N* = 2020) were included in the study using the census method. Figure 1 displays the process of the data selection.

For the purpose of this study, and taking the results of previous studies on diabetes-associated factors into account [12, 13, 18], demographic (age, sex, education, marital status) and anthropometric characteristics (body mass index), nutrition patterns, physical activity, psychological status, smoking and drug abuse, and noncommunicable disease records, including hypertension, diabetes, cardiovascular diseases, and dyslipidemia, were obtained from the SINA Electronic Health Records of the University.

### Ethical considerations

The Ethics Committee of Mashhad University of Medical Sciences reviewed and approved the proposal for carrying out this research with the code of IR.MUMS.FHMPM.REC.1400.022.

### Data analysis

First, the data were descriptively analyzed, and then inferential analysis was performed using Group-Based Trajectory Modeling (GBTM). Based on the Bayesian Information Criterion (BIC), Akaike Information Criterion (AIC), and entropy, the number of trajectories was determined, and groups were added until each trajectory included at least 5% of the sample with the lowest BIC and AIC criteria. In addition to these criteria, to determine the number of classes, the context and objectives of the study and clinical relevance were taken into account [9]. The GBTM identified longitudinal distinct trajectories and subgroups of individuals with a specific pattern of change in HbA1c with the passage of age. Then, to determine the characteristics of members of these subgroups and the factors associated with this grouping, multinomial regression was used to show the relative risk factor at a 0.95 confidence interval. In the present study, the significance level for all tests was 0.05.

## Results

The participants in the study were 2020 people with an age range of 18–59 years. They were divided into two age groups of 18–29 and 30–59, where the latter included 1744 people (86.3%). Women formed 71.3% (*N* = 1441) of the participants. In terms of education, most people, i.e., 47% (*N* = 938), had elementary education. Most of the participants (69%) were housewives and had no income. The body mass index (BMI) of around one-third of the participants (32%) was normal, around one-third were overweight (35%), and around one-third were obese (30%). Moreover, 53% of the participants had good physical activity. Approximately two-thirds of those surveyed did not have hypertension but had dyslipidemia, and more than 90% had no history of cardiovascular diseases. Only 4.16% of the participants smoked or had drug abuse, the majority of whom (91.6%) were in the 30–59 age group.

Dislipidemia was defined as an abnormal level of any of the four variables of triglyceride, LDL, HDL, and cholesterol. The normal levels of these variables for each age group were determined based on medical guidelines [19].

Table 1 displays the existence of dyslipidemia in the two age groups.

**Table 1:** Distribution of dyslipidemia and blood pressure mean by age groups

variable		18–29 year age group	30-59- year age group
Dyslipidemia (n (%))			
	Yes	129 (46.7)	1158 (66.4)
	No	138 (50.0)	582 (33.4)
	Missing	9 (3.3)	4 (0.2)
	Total	276 (100.0)	1744 (100.0)
Blood pressure (mean(SD))			
	Systolic (mmHg)	104.24 (17.22)	115.35 (19.80)
	Diastolic (mmHg)	64.67 (12.27_	71.33 (12.69)

To identify the appropriate number of latent classes, we started with 2-group models, testing zero-order, linear, quadratic, and cubic specifications for the trajectory polynomial shape. Bayesian and Akaike information criteria, entropy index, and clinical characteristics of the disease were used to determine the optimal number of latent class trajectories. The shape of trajectory 2 2 3 2 with a Bayesian information criterion (BIC) of -22238.83, Akaike information criterion (AIC) of -22191.13, and entropy index of 0.70 were selected.

**Fig. 2:**
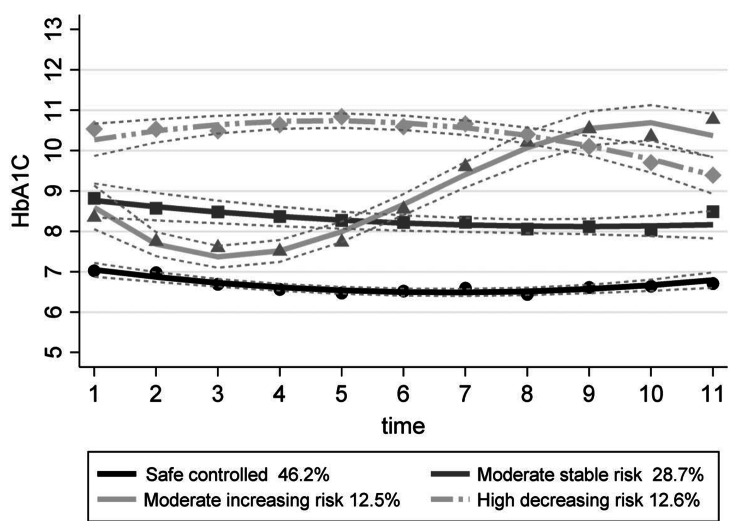
HbA1c trajectories for the four identified groups

Using comparative criteria of information and the entropy index and based on glycosylated hemoglobin levels over time, four trajectories were identified: safe controlled trajectory, moderate stable risk trajectory, moderate increasing risk trajectory, and high decreasing risk trajectory (Fig. 2). Each of these trajectories included at least 5% of all participants. The safe controlled trajectory included people whose glycosylated hemoglobin level over time was approximately 7%, and the moderate risk trajectory included people whose glycosylated hemoglobin level over time was approximately 8.5%. Table 2 shows the number and percentage of people in each trajectory.

**Table 2:** Frequency distribution of latent class trajectory groups

Glycosylated hemoglobin trajectory groups	*N*	%
Safe controlled	933	46.2
Moderate stable risk	580	28.7
Moderate increasing risk	252	12.5
High decreasing risk	255	12.6
Total	2020	100.0

**Table 3:** Average posterior probability (APP) and confidence interval (CI) for the membership of individuals in the identified metabolic control trajectories

	Safe controlled	‌ Moderate stable risk	Moderate increasing risk	High decreasing risk
APP	0.88 (0.88-0.89)	0.74 (0.73-0.76)	0.74 (0.72-0.77)	0.85 (0.83-0.87)

As Table 3 displays, the average posterior probability (APP) in all trajectories is more than 0.7, which indicates that individuals are correctly placed in the metabolic control trajectories.

After determining the optimal number of groups and the shape of the trajectories, multinomial logistic regression was used to investigate the factors associated with these trajectories.

**Table 4:** Factors associated with identified risk trajectories using a multinomial logistic regression model

Factors associated with moderate stable risk trajectory											
Variable		RRR				*p*		SE	z	CI	
Physical activity (No)		1.27				< 0.001		0.14	2.15	1.02–1.58	
Psychological status (Poor)		2.04				< 0.001		0.35	4.14	1.45–2.87	
Dyslipidemia (Yes)		1.96				< 0.001		0.24	5.48	1.54–2.50	
Education (Diploma and high school)		1.46				< 0.05		0.25	2.15	1.03–2.07	
Age (30–59 years)		1.09				> 0.05		0.21	0.46	0.74-1.59	
Smoking and drug abuse (Yes)		1.23				> 0.05		0.34	0.75	0.71-2.14	
constant		0.26				< 0.001		0.09	-3.85	0.13-0.51	
**Factors associated with moderate increasing risk trajectory**											
**Variable**		**RRR**				**p**		**SE**	**z**	**CI**	
Dyslipidemia (Yes)		1.40				< 0.05		0.24	1.97	1.00-1.97	
BMI (Overweight)		0.64				< 0.05		0.13	-2.13	0.43-0.96	
Age (30–59 years)		1.63				> 0.05		0.53	1.50	0.86-3.08	
Smoking and drug abuse (Yes)		1.61				> 0.05		0.56	1.35	0.80-3.22	
constant		0.20				< 0.001		0.11	-2.92	0.07-0.59	
**Factors associated with high decreasing risk trajectory**											
**Variable**		**RRR**				**p**		**SE**	**z**	**CI**	
Sex (Woman)		0.34				< 0.001		0.13	-2.74	0.15-0.73	
Job (Employee)		0.14				< 0.001		0.07	-3.89	0.05-0.38	
Education (Diploma and high school)		1.66				< 0.05		0.41	2.05	1.02–2.71	
BMI (Obese)		0.57				< 0.001		0.12	-2.61	0.38-0.87	
Physical activity (No)		1.67				< 0.001		0.26	3.25	1.22–2.27	
Psychological status (Poor)		3.25				< 0.001		0.67	5.66	2.16–4.90	
Dyslipidemia (Yes)		2.80				< 0.001		0.51	5.59	1.95–4.01	
Age (30–59 years)		1.07				> 0.05		0.28	0.26	0.64-1.79	
Smoking and drug abuse (Yes)		1.64				> 0.05		0.57	1.42	0.82-3.27	
constant		0.20				< 0.001		0.09	-3.32	0.07-0.52	
**Base group (Safe controlled trajectory)** RRR = 1											

The safe controlled group is the base group in this model, the relative risk ratio of which is considered to be 1 for all variables, and the other three groups or trajectories are compared with this base trajectory. Factors associated with the placement of people in the other three trajectories are displayed in Table 4.

According to the values in Table 4, physical inactivity, poor psychological status, dyslipidemia, and education were significantly associated with being in the moderate stable risk group (*p* < 0.05), while there was no significant association between age and smoking and placement on any of the trajectories.

More precisely, according to the value of the relative risk ratio (RRR), if the other variables are kept constant, the probability of placing people without physical activity in the moderate stable risk group is 1.27 times higher than those with physical activity (*p* < 0.001). Poor psychological status also boosts the chance of individuals being in the moderate stable risk group by 2.04 times (*p* < 0.001). People with dyslipidemia were also 1.96 times more likely to be in this group than those without dyslipidemia (*p* < 0.001). People with diplomas and a high school level of education also had 1.46 times higher chance than the illiterate ones to be in this group (< 0.05). In general, it can be said that people without physical activity who have a poor psychological status, dyslipidemia, and high school education have a higher chance of being in the moderate stable risk group.

Table 4 also shows that dyslipidemia and BMI are significantly associated with being in the moderate increasing risk trajectory. Dyslipidemia increased the chances of people being in this group by 1.40 times, and overweight decreased this chance by 0.64 times (*p* < 0.05).

According to Table 4, gender, job, education, BMI, physical inactivity, poor psychological status, and dyslipidemia are significantly associated with being in the high decreasing risk group. The relative risk ratio (RRR) shows that women are less likely to be in the high decreasing risk group (RRR < 1). In other words, men are 2.94 times more likely than women to be in the high decreasing risk group (*p* < 0.001). Examining the row related to the job variable in Table 4 also shows that employees are less likely to be in this group than unemployed people (RRR < 1). To be more precise, if other variables are constant, the chances of unemployed people being in the high decreasing risk group increase by 6.66 times compared to employees and people with administrative jobs (*p* < 0.001). People with a diploma and high school level of education also had 1.66 times higher chance than the illiterate ones to be in this group (< 0.05). Obese people had a lower chance to be in this group (< 0.01). In other words, people with normal weight had 1.75 times more chance than obese people; e to be in this group. Poor physical inactivity can also increase a person’s chance of being in the high decreasing risk group by 1.67 times (*p* < 0.001) and the poor psychological status by up to 3.25 times (*p* < 0.001), and dyslipidemia can increase the chance up to 2.8 times (*p* < 0.001). In short, unemployed men with dyslipidemia and lack of physical activity who are not in a good psychological status are more likely to be in the high decreasing risk group.

## Discussion

As a result of this study, four latent class trajectories were identified, which included the safe controlled group, moderate stable risk, moderate increasing risk, and high decreasing risk. 46% of the participants were in the safe controlled group, and around 28% were in the moderate stable risk group. The other two trajectories each included around 12% of the participants. These results suggest that, as predicted, people with type 1 diabetes are not all homogeneous and take different trajectories in disease control and therefore require attention and intervention appropriate to the type of diabetes control. Among these groups, it seems that the increasing moderate risk trajectory requires more serious follow-up and prevention interventions to take self-care in diabetes control more seriously. After that, the high decreasing risk group also needs special health care attention and follow-up to ensure the continuation of their self-care measures. In other words, given that most patients with type 1 diabetes are in the safe and moderate stable and safe-controlled groups, healthcare providers should focus their time and attention on the other two groups at higher risk.

The results of multinomial logistic regression show that people with high school education who have little physical activity poor psychological states and dyslipidemia are more likely to be in the stable moderate risk group. People with dyslipidemia are more likely to be in the moderate increasing risk group. Normal weight unemployed men with dyslipidemia, poor physical activity, and psychological status who have high school education are more likely to be in the high decreasing risk group.

The findings of the study in different groups show that physical inactivity, poor psychological status, and dyslipidemia, which are known risk factors of diabetes, are factors related to the placement of individuals in different trajectories of metabolic control of diabetes. In addition, unemployment increases a person’s chance of being in the high decreasing risk groups. Unemployment can influence the income of the family, and therefore can probably affect the diet and nutrition of the people. The finding that a normal weight increases a person’s chance of being in the high decreasing risk group is in line with the information that people with type 1 diabetes typically have a normal weight and people who are overweight or obese develop type 2 diabetes.

In terms of comparing the findings of this study with the findings of previous studies, the finding of the present study that gender is effective in metabolic control trajectories is not in line with the findings of Schwant and colleagues [9]. However, the findings of this study on the effectiveness of body mass index and physical activity on the placement of individuals in metabolic control pathways are consistent with their findings. The effect of body mass index on metabolic control is somewhat consistent with the findings of Thomas and colleagues [10] because they found that the body mass index of people with type 1 diabetes was lower than the body mass index of people with type 2 diabetes.

Bigdeli and colleagues [11], in the study of factors affecting self-care behaviors, did not report a significant effect of age, gender, and education, which is consistent with some findings of this study (ineffectiveness of age) and inconsistent with some others (the effect of gender and education). In addition, their finding of a significant negative relationship between obesity and self-care behaviors is consistent with the findings of this study that people with normal weight are more likely to be on the high decreasing trajectory.

The results of our study also showed smoking and drug abuse were not significantly associated with the placement on any trajectories. The reason may be that very few participants reported smoking or drug abuse. This finding of ours is in line with the finding of Cichosz et al. [20], who reported no difference between fasting glucose in smokers, nonsmokers, and ex-smokers. Majority of our study samples were women and this was related to low smoking prevalence. Considering that the population under study includes people who were under active care, so the prevalence of risk factors in them can be lower than the general population. In another studies smoking was associated with inadequate glycemic control [21].

Esteghamati and colleagues [12], in a study conducted in 2019, also reported gender and blood lipids as factors affecting poor blood sugar control. Their findings are consistent with the findings of this study on the effects of gender and dyslipidemia.

Charleer and colleagues’ study [18] also showed that after 24 months of follow-up, HbA_1c_ levels began to decrease. Their study demonstrates the importance of longitudinal studies and follow-up of people with diabetes for effective glycemic control. The connection between this point and the findings of the present study is that since it is not possible to follow all diabetic people, the focus of follow-ups should be on people who are on higher risk trajectories, such as moderate increasing and high decreasing risk trajectories.

This study also has some strengths and limitations. The strength is that although many studies in the past have examined the incidence and prevalence of diabetes and its associated risk factors, all domestic studies in Iran are cross-sectional, and the vast majority of foreign studies conducted longitudinally have used standard growth methods to analyze their data; therefore, only one trajectory of change has been estimated for the whole population or the sample under study. Therefore, the differences between the groups who follow that one path remain anonymous and hidden in the sample, and healthcare providers and health experts provide all people with the same recommendations. However, given the importance of blood sugar control and self-care measures in type 1 diabetes, it is necessary to identify and pay attention to unknown subgroups of individuals who may be hidden in the population. The use of latent class analysis makes this possible.

Despite the strengths, the data used in the study were secondary data recorded for other purposes, and in some cases were not complete and had missing. Since the participants were not available, cases with too many missings were excluded. Moreover, the data had been collected from people who had voluntarily gone to primary healthcare centers, and it is possible that some people with different risk factors have avoided referring to such centers and therefore are absent from the sample. Finally, due to the nature of the design of the study and need for adjustment for more unmeasured confounding, causal relationships cannot be drawn from the results.

## Conclusion

The findings indicate that there are four groups or trajectories among the people with diabetes participating in the study, which include the safe controlled group, the moderate stable risk group, the moderate increasing risk group, and the high decreasing risk group. In addition, the variables of physical inactivity, poor psychological status, and dyslipidemia can affect the chance of people being in these groups; therefore, health professionals and caregivers need to plan self-care education programs and appropriate prevention and treatment programs for the patients according to the metabolic control trajectory in which they are.

This research has been performed using the group-based trajectory modeling method, which considers the variance within groups as zero and does not pay attention to within-group differences; therefore, researchers can analyze the data in the future using more advanced statistical methods such as mixed growth modeling that take into account intergroup and intragroup differences. Further studies on the significant risk factors identified in this study can also be useful and important. Confidentiality of the participants’ identities, avoiding prejudice in interpreting the results, and acknowledging all sources are among the ethical considerations in this study.

## Data Availability

The datasets analyzed during the current study are not publicly available due to the fact that they are part of the SINA Electronic Health Records of Mashhad University of Medical Sciences, but are available from the corresponding author on reasonable request.

## Abbreviations

SD: Standard deviation
CI: Confidence interval
SE: Standard errors
LCGM: Latent class growth modeling
GBTM: Group-Based Trajectory Modeling
BIC: Bayesian information criterion
AIC: Akaike information criterion
RRR: Relative risk ratio
APP: Average posterior probability
